# Physical Therapy in the Management of Pelvic Floor Muscles Hypertonia in a Woman with Hereditary Spastic Paraplegia

**DOI:** 10.1155/2014/306028

**Published:** 2014-11-12

**Authors:** Aline Moreira Ribeiro, Cristine Homsi Jorge Ferreira, Elaine Cristine Lemes Mateus-Vasconcelos, Rafael Mendes Moroni, Luciane Maria Oliveira Brito, Luiz Gustavo Oliveira Brito

**Affiliations:** ^1^Rehabilitation Center of Hospital das Clínicas, Ribeirão Preto School of Medicine, University of São Paulo, 14049-900 Ribeirão Preto, SP, Brazil; ^2^Department of Biomechanics, Medicine and Rehabilitation of the Locomotor System, Ribeirão Preto School of Medicine, University of São Paulo, 14049-900 Ribeirão Preto, SP, Brazil; ^3^Barão de Mauá University Center, 14090-180 Ribeirão Preto, SP, Brazil; ^4^Department of Gynecology and Obstetrics, Ribeirão Preto School of Medicine, University of São Paulo, 14049-900 Ribeirão Preto, SP, Brazil; ^5^Federal University of Maranhão, 65080-805 São Luís, MA, Brazil

## Abstract

*Background*. Pelvic floor (PF) hypertonic disorders are a group of conditions that present with muscular hypertonia or spasticity, resulting in a diminished capacity to isolate, contract, and relax the PF. Their presentation includes voiding and sexual dysfunctions, pelvic pain, and constipation. Various factors are associated, such as complicated vaginal birth, muscular injury, scar tissue formation, and neuropathies.* Study Design*. The case of a single patient will be presented, together with the management strategies employed.* Case Description*. A woman with hereditary spastic paraparesis and a history of muscle spasticity and urinary and fecal complaints since childhood. She presented to this institution seeking treatment for pelvic pain, pain during intercourse, constipation, and micturition problems. A physical therapy protocol was developed, with the trial of several treatment modalities.* Outcome*. After some failed attempts, perineal and pelvic floor stretching proved to be very efficacious therapies for this patient's complaint, leading to improved pain during intercourse, constipation, pelvic pain, and urinary stream.* Discussion*. PF spasticity can lead to severe disability and interfere with daily basic functions, such as micturition and evacuation. Physical therapy plays an essential role in the management of these patients and can lead to significant improvement in quality of life.

## 1. Introduction

The pelvic floor (PF) is a neuromuscular structure that supports the pelvic viscera and allows them to function individually. Hypertonic disorders of the pelvic floor are very common and may be associated with several factors, such as recurrent urinary infections, prolonged voiding intervals, and obstetric injuries. They may also be related to chronic pelvic pain and neurologic diseases [1].

Hereditary spastic paraplegia (HSP), or Strumpell-Lorrain disease, is a neurodegenerative disorder characterized by muscle weakness and spasticity in the lower limbs, having an estimated prevalence of 1.27–9.6/100.000 [2]. It is related to degeneration or to a disorder in the development of the pyramidal axis, with hyperexcitability of the stretch reflex. Physical rehabilitation is essential in the management of the spasticity caused by HSP, usually accompanied by other treatment strategies, such as pharmacological treatment, botulinum toxin, local heat, cryotherapy, ultrasound, functional electrical stimulation, biofeedback, and surgery. PF muscle hypertonia, with its diverse etiology, may be managed through different strategies discussed in the literature, but a strategy specifically aimed at managing pelvic floor spasticity caused by neurologic disease was not found. The case report of a woman with pelvic floor hypertonia due to HSP is presented, together with the management strategies employed.

## 2. Study Design

This paper reports the case of a single patient with the diagnosis of Strumpell-Lorrain disease who presented with pelvic pain and urinary and fecal complaints.

## 3. Case Report

J. C. S., a 34-year-old nullipara, started treatment at this institution during childhood, in 1981, due to epileptic seizures and episodic loss of consciousness, which were followed by gaiting difficulties, due to the fact that her toes could not be extended, limitations to perform daily activities, frequent falls, intestinal constipation, pain with bladder filling, and urgency urinary incontinence. Her father, brother, and sister had a similar history, leading to the diagnosis of HSP after extensive clinical investigation and to initial treatment with gabapentin.

She was referred to the urogynecology division in this hospital in 2011, due to dysuria, incomplete bladder emptying, and the need to use abdominal effort during micturition. She also presented intestinal constipation, with up to 5 days without passing stools, and the need for intense straining to complete evacuation. She had diminished daytime urinary frequency, nocturia, and episodes of nocturnal enuresis. Other complaints were pain during intercourse and urinary/fecal urgency during coitus. Urodynamics were performed and suggested bladder outlet obstruction. She was started on doxazosin and referred to physical therapy for PF muscles rehabilitation.

## 4. Evaluation and Management

During physical therapy evaluation, pelvic floor muscle assessment revealed grade 1 strength, according to the Oxford Scale. There were diffuse tender nodules throughout the pelvic muscles, and the patient complained of pain during digital vaginal palpation. A 24 h bladder diary demonstrated low fluid intake, a low daytime urinary frequency (4 times/day), and nocturia (4 times/night), with no urinary loss. Behavioral therapy was initially tried, focusing on adequate fluid intake and programmed micturitions, based on the filling of bladder diaries.

Afterwards, treatment was started with Thiele's perineal massage, abdominal massage, to address the constipation complaints, and PF muscles contraction exercises during a 10-week period and 1 session/week, comprising 10 sessions. The patient was instructed on adequate posture during micturition and defecation and also to recruit abdominal muscles during these activities, avoiding Valsalva and PF overload during straining. The use of lubricant during coitus was also emphasized.

After a month, she presented a slight improvement in pain during intercourse and a more significant improvement in constipation, with daily bowel movements. Pelvic awareness exercises with the use of a Swiss ball (anteversion, retroversion, and pelvic tilt) were included in the treatment protocol, together with abdominal muscle strengthening. She maintained waxing and waning constipation, without significant improvement in pain during intercourse or urinary complaints.

Due to limited response, the initial treatment was switched to perineal cryotherapy, aiming at spasticity control with weekly sessions during 1 month. Results were also unsatisfactory, due to low patient tolerance and low adherence, and the treatment protocol had to be changed once again.

During reevaluation, she referred to partial relief from pain during intercourse with the adoption of more comfortable positions and with the employment of rapid and smooth pelvic floor muscles contraction during coitus. Based on such information, the use of a biofeedback vaginal probe (Perina, Quark, Brazil), disconnected from the main device and coupled to an insufflation pump, was started during exercises during three months (total of 14 sessions). She was instructed to perform smooth and nonsustained contractions, coupled with muscle stretching through the use of the vaginal probe during muscle relaxation. With this new approach, there was significant improvement in pain during intercourse, urinary stream, and constipation. The use of the squatting position during the exercises was also implemented, allowing for greater muscle stretching. Due to associated lower limb spasticity, the squatting position could only be maintained with the support of balls or benches.

The patient is still in rehabilitation, has relearned urinary and defecatory habits, and presents great improvement in quality of life. Nonetheless, she still experiences some occasional worsening in her complaints, demanding constant reevaluation.

## 5. Discussion

Since there is no well-defined strategy for management of spasticity in PF muscles due to neurologic diseases, the management of this case had to be individualized, literature regarding the management of pelvic floor hypertonia had to be consulted, and several techniques were tried before a satisfactory improvement could be seen.

Initially, the proposed treatment involved perineal massage, described by Thiele in 1937 for patients without genitourinary complaints, but with muscle spasm in the levator ani and coccygeus muscles. Such technique stabilizes trigger points in those muscles, resulting in muscle tension inhibition, stretching, and relaxation [3]. The massage also generates local heat, increases vascularization, and improves muscle tissue mobility [4]. It is recommended that, together with the massage, isometric contractions of the puborectalis muscle be performed [5]. In this patient, Thiele's massage was not able to achieve persistent improvement, so another technique, cryotherapy, was tried. It decreases in conduction speed for neural impulses, both in myelinated and nonmyelinated fibers, of about 2.4 m/s per °C of cooling. Consequently, pain perception and muscle contractility are decreased, muscle spindle response to stretch is decreased, and, as a result, muscle spasticity is controlled [6]. Duration of cryotherapy for effect over pain and muscle spasm is about 12 to 15 minutes [7]. The effect may persist for up to 2 hours and may be used to facilitate kinesiotherapy, since it promotes muscle relaxation. Up to 25–35% of patients, however, may not show improvement with the technique and some may even present worsening of spasticity [8].

There was limited tolerance of the patient to cryotherapy, leading to its discontinuation. Another technique, muscle stretching, was then tried, through the use of the insufflated vaginal probe. Muscle spasm is associated with decreased plasticity due to muscle and connective tissue shortening, secondary to adhesions and scar tissue formation within the muscle. Muscle stretching improves spasticity by increasing local temperature and leading to breaks in the connective tissue sheath overlying the muscles, thereby improving muscle flexibility [9]. To obtain adequate PF muscle stretching, two approaches were used: firstly, through the use of an insufflated vaginal probe, promoting massage and perineal desensitization; secondly, through the adoption of the squatting position during exercises. Magnetic resonance pelvimetry studies in different positions showed larger interspinous, intertuberous, and sagittal diameters in the squatting position compared to other positions studied, promoting pelvic floor stretching [10]. The combination of these two interventions led to maximal PF stretching and to significant improvement in the patient's complaints.

The role of the physical therapist is to stimulate discipline and responsibility of the patient, aiming to be successful in his/her treatment. This health professional must be dynamic, resilient, and flexible with all information that his/her patient brings to him/her every session; this differential will make him/her modify his treatment strategies. In neurologic diseases like this case report, perseverance is demanded of him/her to obtain favorable results in a larger period of time and probably this was the key for the success of this case.

## 6. Conclusions

Although there is a lack of studies on the management of PF muscles hypertonia due to neurologic diseases, the protocol used in this case was effective and well tolerated, highlighting the importance of physical therapy in the rehabilitation of these patients. New trials are needed to validate these findings.
